# cGMP-Dependent Protein Kinase Iβ Interacts with p44/WDR77 to Regulate Androgen Receptor-Driven Gene Expression

**DOI:** 10.1371/journal.pone.0063119

**Published:** 2013-06-03

**Authors:** Liran Zhou, Keiko Hosohata, Shen Gao, Zhongping Gu, Zhengxin Wang

**Affiliations:** Department of Cancer Biology, The University of Texas M. D. Anderson Cancer Center, Houston, Texas, United States of America; University of Salerno, Faculty of Medicine and Surgery, Italy

## Abstract

The androgen receptor (AR) pathway plays critical roles in controlling differentiation and proliferation of prostate epithelial cells. We previously identified a novel AR cofactor, p44/WDR77, which specifically enhances AR transcriptional activity in the prostate gland and prostate cancer. To further elucidate p44/WDR77's role in the AR signaling pathway, we conducted a yeast two-hybrid screening and identified cGMP-dependent protein kinase (PKG) as a p44/WDR77-interacting protein. Further investigation by lusiferase assay and kinase assay demonstrated that PKG-Iβ physically interacted with and phosphorylated both p44 and AR and enhanced AR transactivity in synergy with p44 in an androgen- and cGMP-dependent manner. Furthermore, PKG1β expression promoted p44/WDR77 nuclear translocation and inhibited prostate cancer cell growth via G1 cell cycle arrest. Our findings characterize PKG as a novel regulator of AR-mediated transcription by enhancing AR cofactor p44/WDR77's function, which provide a novel mechanism for the growth regulation of prostate cancer cells by the androgen signaling.

## Introduction

Androgens play versatile roles in regulating the survival, development, growth, and differentiation of the prostate gland [1]. Androgens' biological functions are mediated by androgen receptor (AR), a steroid nuclear receptor [2], [3]. The AR signaling pathway also plays critical roles in prostate cancer initiation and progression [4]–[6]. As a ligand-activated transcription factor, AR translocates from the cytoplasm to the nucleus upon androgens binding, recognizes the androgen response element (ARE) of target genes, and recruits cofactors to regulate target gene expression [7]–[10].

We purified and cloned a novel AR-interacting protein (p44), which regulates expression of a subset of AR-target genes in the prostate gland as well as in prostate cancer [11]–[14]. P44 is composed of 342 amino acid residues and 7 putative WD-40 repeats [13] and is designated as the WD Repeat Domain 77 (WDR77) in the Gene database (www.ncbi.nlm.nih.gov/gene/79084). The p44 protein localizes in the cytoplasm of prostate epithelial cells at the early stage of prostate development, when epithelial cells are rapidly proliferating [13]–[15]. In contrast, p44 localizes in the nucleus of adult prostate epithelial cells, that are fully differentiated and not dividing. P44 in the cytoplasm is essential for growth of prostate epithelial cells and p44 in the nucleus is required for functional differentiation of luminal cells occurring with the expression of the prostate-specific secretory proteins [16]–[19]. The *p44*-*null* prostate was small and not fully differentiated and was deficient in production of secretory proteins [11].

The p44 expression was examined in matched prostate cancerous and benign prostate tissues derived from 44 patients with prostate cancer [13]. The p44 immunostaining signal was strong in the nuclei of epithelial cells in the benign areas, but absent in the stroma cells. In contrast, in the tumor areas, the nuclear staining was significantly decreased and strong p44 immunostaining was observed in the cytoplasm of cancer cells. Similarly to prostate cancer samples, p44 localized in the cytoplasm of prostate cancer LNCaP, 22RV1, PC3, and DU145 cells [20]. When selectively expressed in the nucleus by fusing a strong nuclear localization signal (NLS) at the N-terminus of the p44 protein, the nuclear p44 strongly inhibited growth of prostate cancer cells in the tissue culture and of prostate tumors in nude mice to arrest the cell cycle at the G1/G0 phase [13], [14].

The prostate gland is enlarged with aging [21]–[25]. Prostatic intraepithelial neoplasia (PIN) is generally detected in the growing prostate in the aged men and accepted as a premalignant lesion that has potential to progress to prostate cancer [26]–[29]. The age-related growth of the prostate is a critical step leading to abnormal proliferation and tumorigenesis [24], [25]. Very little is known about what regulates this age-related growth of the prostate. The p44 protein localizes in the nucleus of benign epithelial cells in the prostate and the p44 cytoplasm translocation is associated with the age-related PIN (from one layer to two and multiple layers of cells) [13]. But, the signal controls this translocation event is not clear.

These studies identify p44 as a factor regulates the transition from cellular proliferation to differentiation through its subcellular translocation [13]–[15]. P44 in the cytoplasm of epithelial cell at early stage of prostate development is required for cell growth and in the adult prostate, p44 in the nucleus establishes and maintains luminal epithelia in a growth-arrested fully differentiated state (the G1/G_0_ cell cycle phase). In the aging prostate, p44 is transported into the cytoplasm and as a consequence, the nuclear p44-mediated growth arrest is relieved and prostate epithelial cell proliferation is initiated by the cytoplasmic p44.

cGMP-dependent protein kinase (PKG) is a serine/threonine-specific protein kinase that is widely distributed in eukaryotes. PKG mediates the cGMP signaling in many biological processes, including smooth muscle regulation [30]–[32], vascular signaling [33], and germ cell development [34]. PKG is composed of two major function domains: regulatory domain including an N-terminal domain (NTD) that interacts with PKG substrates and mediates homodimerization and autoinhibition [35]–[37] and a cGMP binding domain with two non-identical cGMP-binding sites [38]; and kinase domain (KD) including an ATP-binding domain that catalyzes ATP hydrolysis and a catalytic domain (CD). In the absence of cGMP, PKG is autoinhibited by interactions between amino acid residues in its CD and the substrate-like sequences (*e.g.*, RRXSX and RRXAX) in its regulatory domain [35]–[37]. The binding of cGMP to PKG's regulatory domain leads to a conformational change that results in the release of the catalytic core from the NTD, which enables the phosphorylation of substrate proteins [35]–[37].

Two encoding PKG genes, type I (PKG-I) and type II (PKG-II), have been identified in mammals and are highly conserved in kinase domains [39]. PKG-I is predominantly localized in the cytoplasm and translocates into the cell nucleus when stimulated by cGMP [40], while PKG-II is anchored to the plasma membrane by N-terminal myristylation [39]. One of PKG-I's isoforms, PKG-Iβ, has been shown to be involved in transcriptional regulation by directly interacting with transcription factor (TF) II-I [41], [42] and activating the *c-fos* promoter via several promoter response elements, including serum response element (SRE), activating protein-1 (AP-1) binding site (FAP), and cAMP response element (CRE) [43].

To better understand p44's role in prostate cancer and in the prostate gland, we used a yeast two-hybrid assay with full-length p44 as the bait to screen for proteins involved in p44's regulation of AR-mediated transcription. We identified PKG as a p44-interacting protein and found that PKG-Iβ physically interacts with p44 *in vitro* and *in vivo* and functionally regulates AR transactivity. Furthermore, PKG-Iβ expression promoted p44 nuclear localization and inhibited prostate cancer cell growth via G1 cell cycle arrest.

## Results

### Identification of PKG as a novel p44-interacting protein by yeast two-hybrid screening

**Figure 1:**
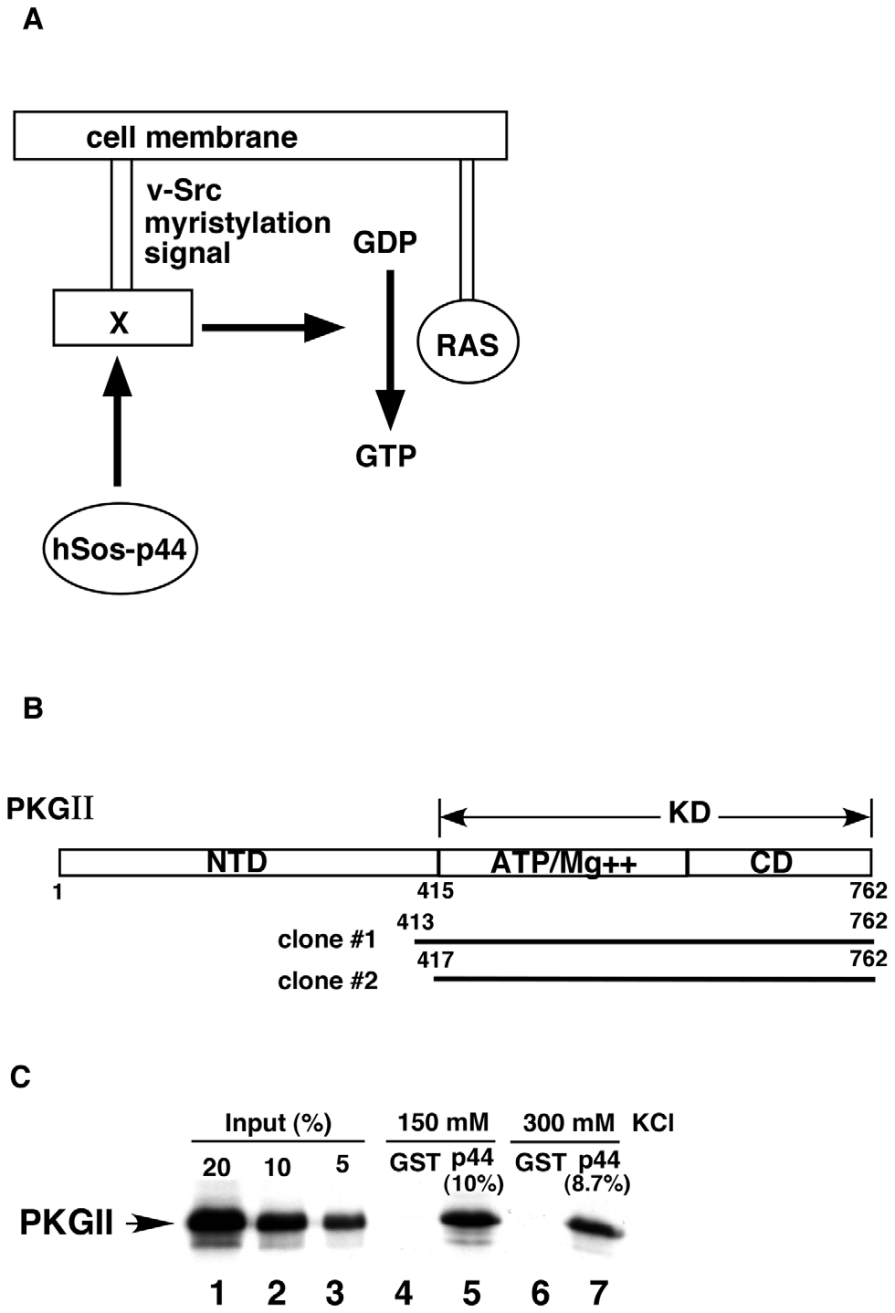
The cGMP-dependent protein kinase (PKG)-II kinase domain (KD) interacts with p44. (A) Diagram of the Ras-signaling pathway utilized in the yeast two-hybrid system. “X” represents the candidate protein that may interact with p44. (B) Diagram of the functional domains of human PKG-II and two clones obtained from yeast two-hybrid screening. The two clones encode 413–762 and 417–762 of PKG-II respectively. (C) The PKG-II KD interacts directly with p44. GST (lanes 4 and 6) or GST-p44 (lanes 5 and 7) fusion protein expressed in bacteria was immobilized on glutathione agarose beads. Beads were incubated with the ^35^S-labeled PKG-II KD in BC150–0.1% (NP)-40 (lanes 4 and 5) or BC300–0.1% NP-40 (lanes 6 and 7) for 2 h at 4 C. After being washed with the incubation buffer, the beads were boiled with SDS sample buffer and analyzed by SDS-PAGE followed by autoradiography. Lanes 1–3 are 20%, 10%, and 5% inputs of lysate used for the interaction assay. NTD, N-terminal domain; CD, catalytic domain.

To identify the mechanisms by which p44 regulates AR-dependent transcription, we performed a yeast two-hybrid assay to screen for p44-interacting proteins (Fig. 1A). We screened approximately 4 million prostate cDNA library transformants and obtained 28 positive clones. Two independent clones encode the human PKG-II KD (Fig. 1B). We confirmed the protein-protein interaction between the PKG-II KD (amino acid residues 415–762) and p44 using a glutathione S-transferase (GST)-fusion protein pull-down assay. We found that the PKG-II KD strongly bound to GST-p44 (Fig. 1C, lane 5), though this interaction was resistant to a high salt concentration (Fig. 1C, lane 7). We did not observe any interactions between the ^35^S-labeled PKG-II KD and GST (Fig. 1C, lanes 4 and 6), indicating the specificity of the p44-PKG-II KD interaction.

### PKG-Iβ enhances AR-dependent transcription

**Figure 2:**
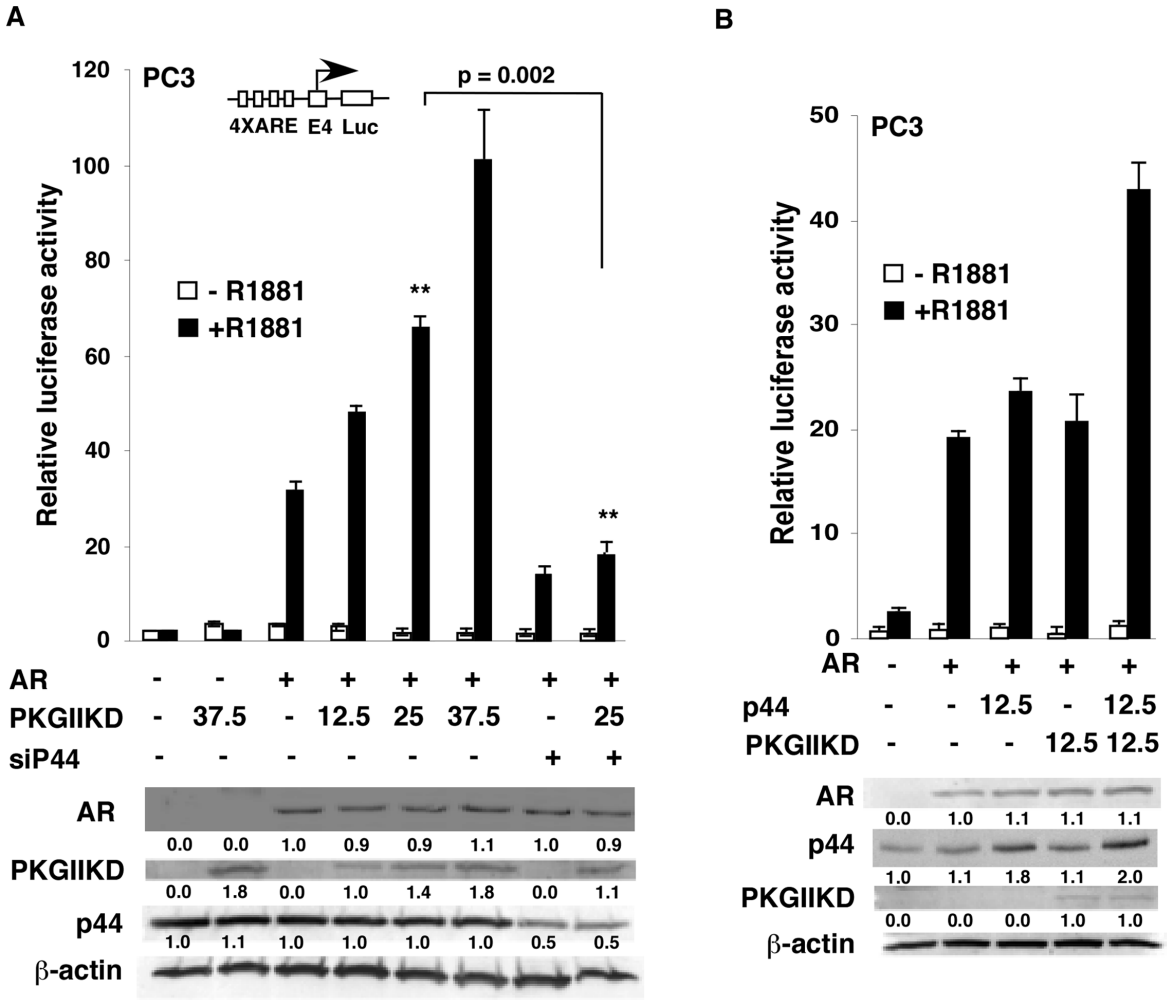
The cGMP-dependent protein kinase (PKG)-II kinase domain (KD) synergizes with p44 to enhance androgen receptor (AR)-driven gene expression. (A) The PKG-II KD enhances AR-mediated transcription. PC3 cells were transfected with 25 fmol of 4× androgen response element (ARE)-E4-luciferase reporter, 2.5 fmol of pcDNA-AR, and the indicated amounts (fmol) of pcDNA-PKG-II KD. Cells were grown in the absence or presence of 10 nM R1881 for 48 h after transfection and then harvested for the luciferase activity assay. (B) The PKG-II KD synergizes with p44 to increase AR-driven gene expression. PC3 cells were transfected with 25 fmol ARE-E4-luciferase reporter, 0.8 fmol pcDNA-AR, 12.5 fmol pcDNA-p44, and 12.5 fmol pcDNA-PKG-II KD. Cells were grown in the absence or presence of 10 nM R1881 for 48 h after transfection and then harvested for dual luciferase activity assay. The values represent the mean ± SD in the luciferase assays (n = 3). The bottom panels show Western blot of protein lysates of transfected cells with anti-bodies as indicated. The underneath values represent relative expression of the given protein.

Based on our previous observation that p44 is an AR cofactor and our current finding that the PKG-II KD is a p44-interacting protein, we tested whether the PKG-II KD is involved in AR-mediated transcription. We employed a luciferase report system containing one luciferase reporter driven by androgen response element (ARE) and one AR expression vector. The system was transfected into PC3 human prostate cancer cells. AR activated the reporter activity about tenfold in the presence of synthetic androgen R1881 but did not activate reporter activity in the absence of the androgen (Fig. 2A). PKG-II KD coexpression further enhanced this activity in a dose-dependent manner in the presence of androgen. In contrast, the PKG-II KD did not affect luciferase reporter activity in the absence of AR or the androgen, confirming that the activation of AR target element by PKG-II KD is through androgen-activated AR. Silence of P44 expression via siRNAs significantly decreased AR transactivation as well as the PKGIIKD-mediated enhancement of the report activity (Fig. 2A), indicating that p44 mediates effect of PKGIIKD on androgen-driven transcription.

Next, we investigated whether the PKG-II KD regulates AR activity independently or synergistically with p44, and we cotransfected the luciferase report system with PKG-II KD and p44 into PC3 cells. We found that low amounts (12.5 fmoles) of the PKG-II KD or p44 alone had little effect on AR-dependent transcription (Fig. 2B). However, combining PKG-II KD with p44 resulted in 2.5-fold activation, indicating that the PKG-II KD and p44 regulate AR-driven transcription synergistically.

**Figure 3:**
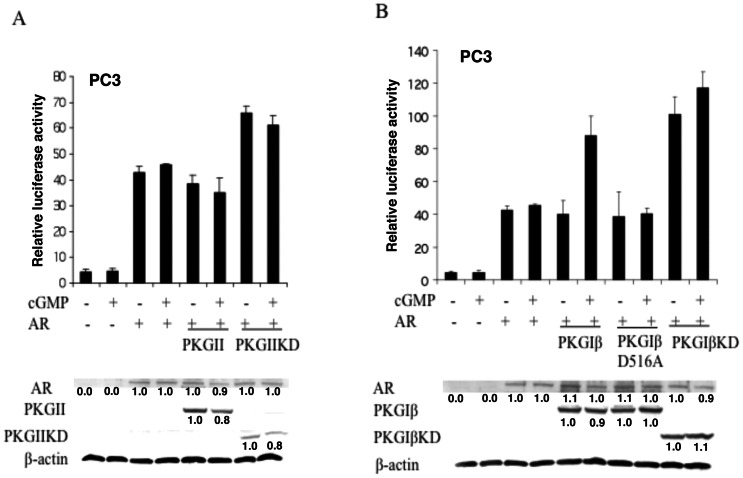
cGMP-dependent protein kinase (PKG)-Iβ enhances androgen receptor (AR)-mediated transcription. (A, B) PC3 cells were transfected with 25 fmol of androgen response element ARE-E4-luciferase reporter, 0.8 fmol of pcDNA-AR, 12.5 fmol of pcDNA-p44, and 37.5 fmol of wild-type pcDNA-PKG-1β, mutant pcDNA-PKG-1β (D516A), or pcDNA-PKG-II. Cells were grown in the presence of 10 nM R1881 for 48 h after transfection. cGMP (1 mM) was added to the medium 4 h before cells were harvested for the dual luciferase activity assay. The bottom panels show Western blot of protein lysates of transfected cells with anti-bodies as indicated. The underneath values represent relative expression of the given protein.

Because the KDs of PKG-I and PKG-II are highly conserved (75% similarity) [39], we tested whether full-length PKG-I or PKG-II regulated AR-dependent transcription. PC3 cells were transfected with the luciferase report system and PKG-Iβ or PKG-II with or without R1881 or 8-Br-cGMP. Surprisingly, full-length PKG-II had no effect on AR-driven transcription (Fig. 3A) while wild-type PKG-Iβ enhanced AR transactivation in the presence of cGMP (Fig. 3B). This observation is consistent with previous observations that PKG-I regulates gene transcription by shuttling between the cell cytoplasm and nucleus, while PKG-II is anchored to the plasma membrane to mediate signal transduction [39]. Therefore, we focused on PKG-Iβ, not PKG-II, to specify PKG's role in AR-mediated transcription. The regulation of AR activity by PKG-Iβ was abrogated by the mutation (D516A) which abolishes the kinase activity of PKG-Iβ [40], indicating that the kinase activity is crucial for PKG-Iβ to regulates AR-driven transcription.

### PKG-Iβ physically interacts with and phosphorylates p44 and AR

**Figure 4:**
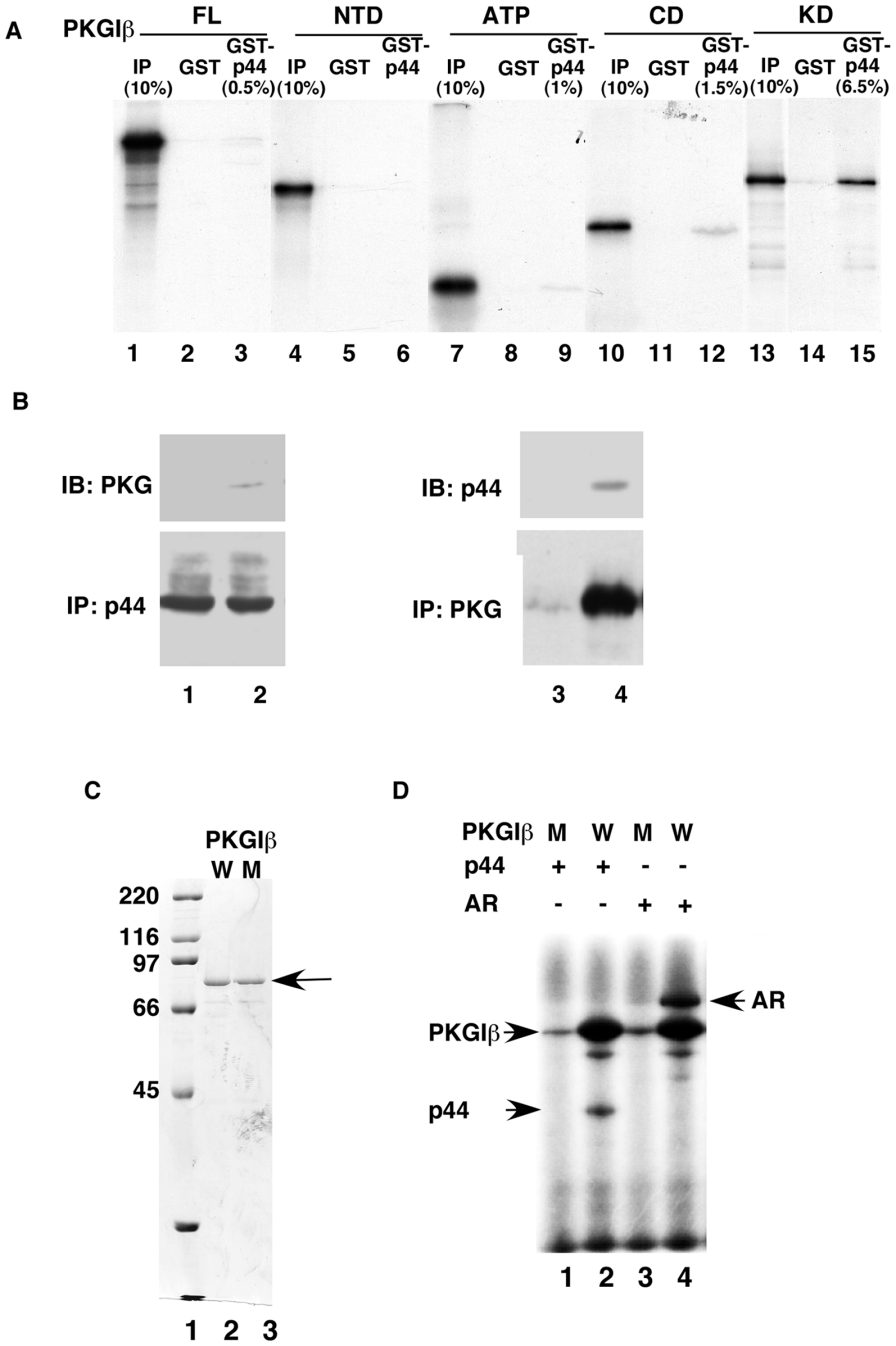
cGMP-dependent protein kinase (PKG)-Iβ physically interacts with p44. (A) PKG-Iβ interacts with p44 *in vitro*. S^35^-labeled full-length PKG-Iβ (FL; amino acid residues 1–686) and the N-terminal domain (NTD; amino acid residues 1–355), ATP-binding domain (ATP; amino acid residues 355–491), catalytic domain (CD; amino acid residues 491–686), and kinase domain (KD; amino acid residues 355–686) of PKG-Iβ were incubated with GST (lanes 2, 5, 8, 11, and 14) or GST-p44 agarose beads (lanes 3, 6, 9, 12, and 15). autoradiography. 10% or 5% input (IP) of lysate used for the interaction assay were loaded onto the gels. (B) PKG-Iβ interacts with p44 in LNCaP cells. LNCaP cells were transfected with pcDNA (lanes 1, 3) or pcDNAf:PKG-Iβ with (lanes 2 and 4). Whole-cell extracts were prepared from the transfected cells and incubated with 10 µl of anti-p44 antibody immobilized on Protein A Sepharose beads (lanes 1, 2) or M2 agarose beads (lanes 3, 4). The immunoprecipitated proteins were analyzed by Western blot with an anti-PKG or -p44 antibody as indicated. (C) SDS-PAGE analysis of recombinant PKG-Iβ. Lanes 2 and 3 show 100 µg of recombinant wild-type (WT) and mutant (M; D516) PKG-Iβ, respectively. The arrow indicates the band that corresponds to PKG-Iβ. (D) PKG-Iβ phosphorylates AR and p44. Wild-type or mutant (D516A) PKG-Iβ were incubated with 80 ng of purified AR or 40 ng of purified p44 and 10 µCi of γ-^32^P-ATP for 1 h at 30 C. Arrows indicate the bands that correspond to phosphorylated AR, PKG-Iβ, and p44.

To identify the domain in which PKG-Iβ interacts with p44, we generated DNA constructs expressing the various domains of PKG-Iβ to detect the interaction of these domains with p44. GST pull-down assay showed that full-length PKG-Iβ weakly interacted with p44 (Fig. 4A, lane 3). The ATP-binding domain (ATP) and C-terminal domain (CD) of PKG-Iβ interacted with p44 (Fig. 4A, lane 9 and 12); however, the PKG-Iβ N-terminal domain (NTD) did not interact with p44 (Fig. 4A, lane 6). Similar to the PKG-II kinase domain (KD), the PKG-Iβ KD interacted strongly with p44 (Fig. 4A, lane 15), suggesting that p44 may be a substrate of PKG-Iβ.

To investigate the interaction of PKG-Iβ and p44 *in vivo*, we performed a coimmunoprecipitation experiment with anti-p44 antibody using LNCaP cells that were transfected with FLAG-tagged PKG-Iβ. PKG-Iβ weakly interacted with p44 (Fig. 4B, lane 2). As a negative control, no PKG was immunoprecipitated from the LNCaP lysate transfected with empty vector (pcDNA3.1) (Fig. 4B, lane 1). Similar coimmunoprecipitation analysis with anti-FLAG antibody also indicated that PKG-Iβ physically interacted with p44 in LNCaP cells (Fig. 4B, lanes 3, 4).

Because p44 physically interacted with PKG-Iβ KD, we postulated that p44 is a substrate of PKG-Iβ. We performed an *in vitro* kinase assay to investigate whether PKG-Iβ phosphorylates p44. The wild-type and kinase-deficient D516A mutant PKG-1β were expressed in and purified from PC3 cells (Fig. 4C, lanes 2 and 3). PKG-Iβ phosphorylated both p44 and AR (Fig. 4D, lanes 2, 4). As indicated by the diminished phosphorylation of p44 and AR with kinase-deficient PKG-Iβ (Fig. 4D, lanes 1, 3), PKG-Iβ's kinase activity was essential for enabling PKG-Iβ's phosphorylating function.

### PKG-Iβ specifically enhances AR-, GR-, and PR-dependent transcription

**Figure 5:**
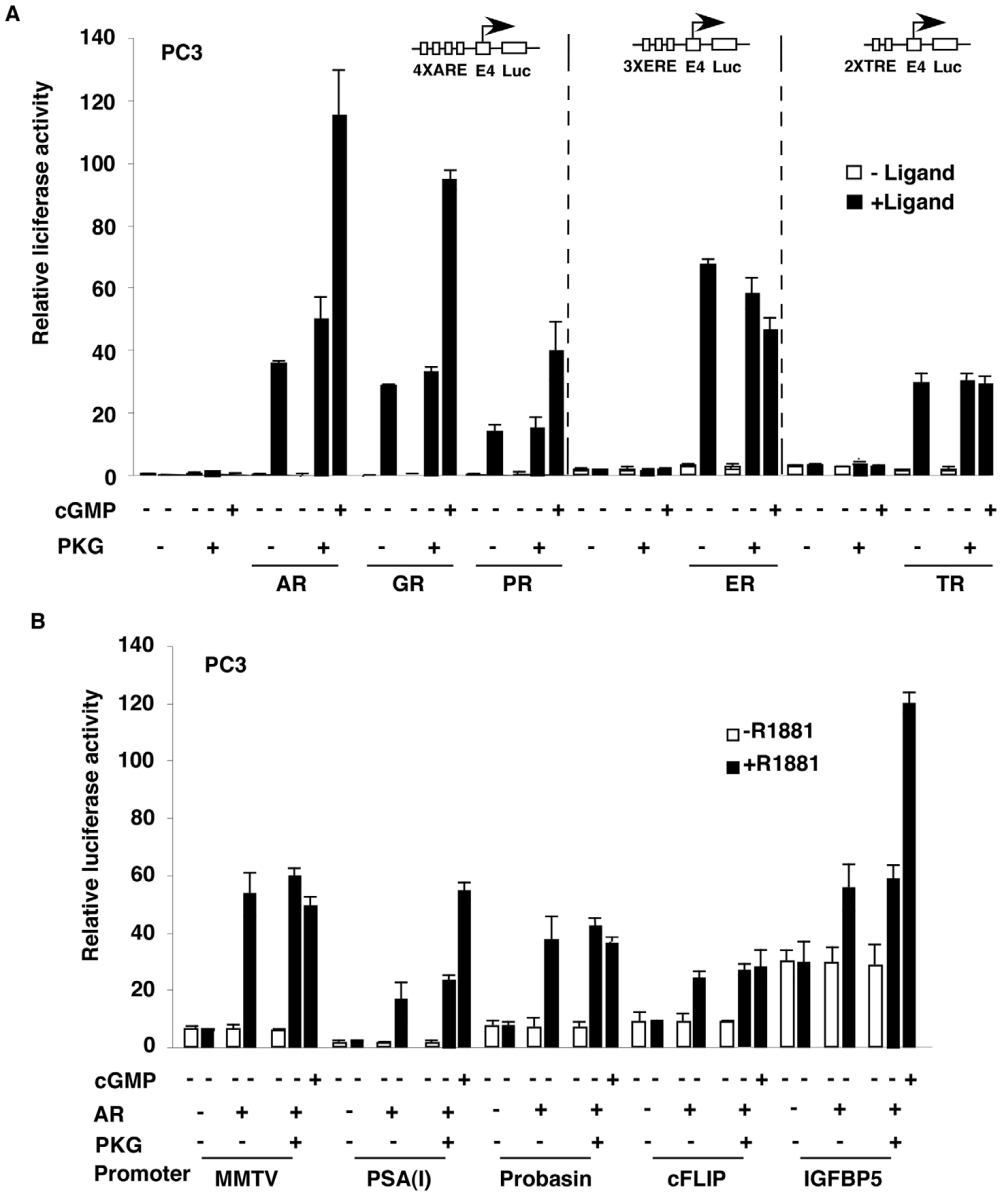
PKG-Iβ affects AR-, GR-, and PR-driven promoter activity. (A) cGMP-dependent protein kinase (PKG)-Iβ specifically enhances androgen receptor (AR)-, glucocorticoid receptor (GR)-, and progesterone receptor (PR)-dependent transcription. PC3 cells were transfected with 25 fmol of 4× androgen response element (ARE)-, 3× estrogen response element (ERE)-, or 2× thyroid response element (TRE)-E4-luciferase reporter; 8 fmol of pcDNA-AR, -GR, -PR, -estrogen receptor (ER), or -thyroid receptor (TR); and 37.5 fmol of pcDNA-PKG-Iβ kinase domain (KD) expression plasmid. Cells were grown with or without 10 nM R1881, 10 nM dexamethasone, 10 nM progesterone, 1 µM estradiol, 10 nM T_3,_ or 1 mM cGMP as indicated for 48 h after transfection and then harvested for luciferase activity assays. (B) cGMP-Dependent Protein Kinase (PKG)-Iβ Selectively Affects Androgen Receptor (AR)-Mediated Gene Expression from Different Promoters. PC3 cells were transfected with 25 fmol of mouse mammary tumor virus (MMTV), PSA enhancer (PSA[I]; −4354/–3858), probasin (−2441/+28), cellular Fas/FasL-associated death domain protein-like inhibitory protein (cFLIP; −48/+156), and IGF binding protein 5 (IGFBP5; −98/+148) luciferase reporters; 2.5 fmol of pcDNA-AR; and 37.5 fmol of pcDNA-PKG-Iβ kinase domain (KD). Cells were grown in the presence or absence of 10 nM R1881 and 1 mM cGMP as indicated for 48 h after transfection and then harvested for luciferase activity assays.

To clarify whether PKG-Iβ specifically regulate AR-mediated transcription, we investigated the effects of PKG-Iβ on transcriptions under the control of glucocorticoid receptor (GR), progesterone receptor (PR), estrogen receptor α (ERα), and thyroid receptor (TR). Similar to the AR, the PKG-Iβ also strongly enhanced GR and PR transactivity in an 8-Br-cGMP- (Fig. 5A) and ligand-dependent manner, though it did not affect ERα- or TR-mediated transcription. Thus, PKG-Iβ selectively enhanced the transcriptional activity of a nuclear receptors subfamily, including AR, GR and PR.

### PKG-Iβ affects AR-driven promoter activity selectively

Based on the observation that PKG-Iβ enhanced AR's transactivity on the synthetic ARE-containing promoter, we investigated PKG-Iβ's effect on natural AR-driven promoters. Plasmids containing AR-response reporters derived from mouse mammary tumor virus (MMTV), PSA enhancer [PSA(I)], probasin, cellular Fas/FasL-associated death domain protein-like inhibitory protein (c-FLIP), and IGF-binding protein 5 (IGFBP5) were subjected to transient transfection and luciferase assay (Fig. 5B). Although it had almost no effect on MMTV, probasin, or c-FLIP promoters, the PKG-Iβ enhanced AR activity on the transcription of PSA enhancer [PSA(I)] and IGFBP5 in the presence of the androgen, evidence that PKG-Iβ has specificity in AR target genes.

### PKG-Iβ enhances AR-, PR-, and GR-dependent gene expression *in vivo*

**Figure 6:**
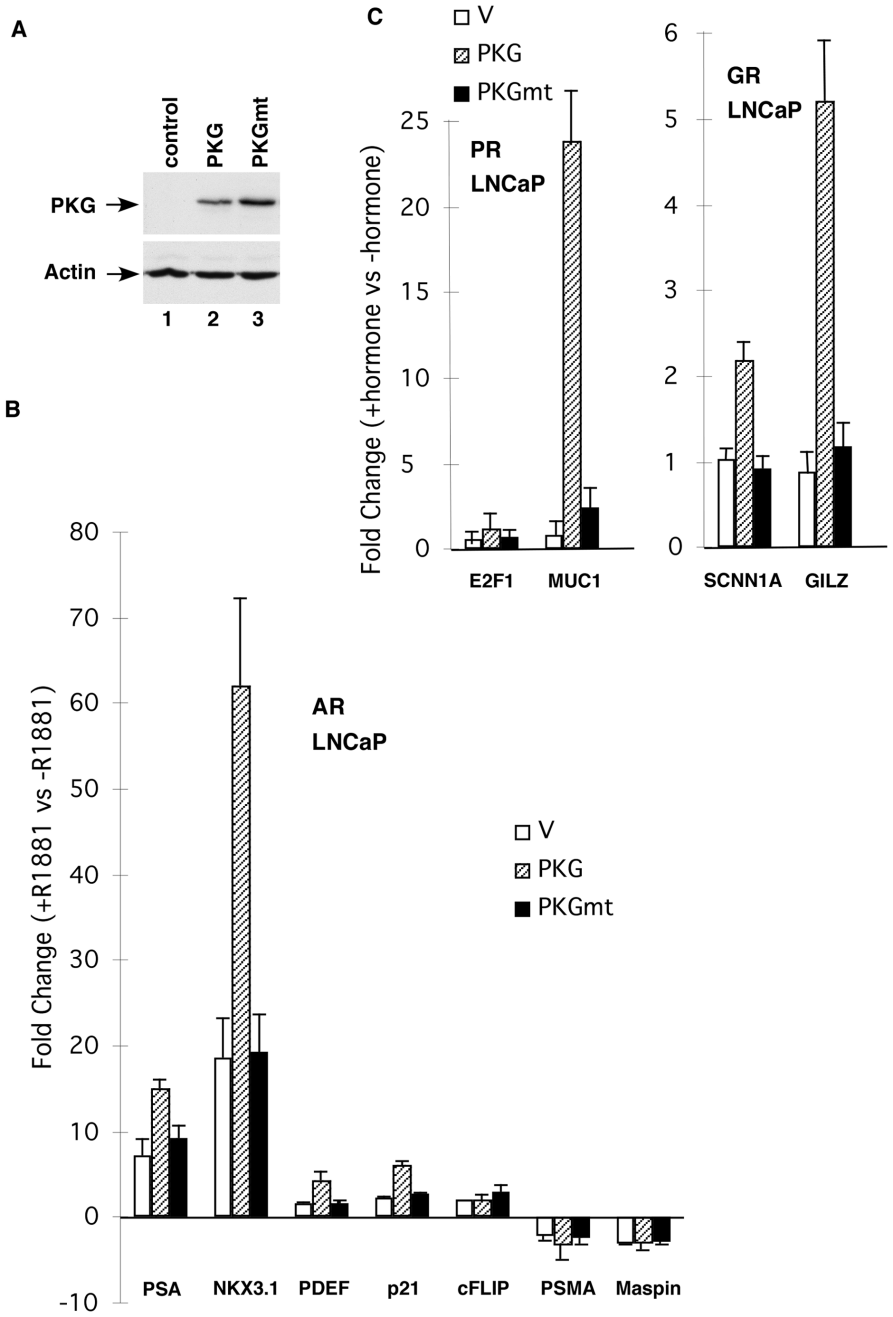
cGMP-dependent protein kinase (PKG)-Iβ selectively affects androgen receptor (AR)-dependent gene expression *in vivo*. (A) Western blot analysis of the expression of wild-type PKG-Iβ or D516A mutant PKG-Iβ in LNCaP cells. Whole-cell lysates (20 µg) were prepared from LNCaP cells expressing the wild-type PKG-Iβ (lane 2) or mutant PKG-Iβ (lane 3) and analyzed with Western blot analysis with anti-PKG antibody. (B) Real-time RT-PCR analysis of AR-target gene expression in LNCaP cells expressing the wild-type PKG-Iβ or mutant PKG-Iβ. The expression of AR target genes in cell lines treated with or without 10 nM of R1881 genes was determined by real-time RT-PCR, and the fold change  =  gene expression in the presence of R1881/gene expression in the absence of R1881. PSA, prostate-specific antigen; PDEF, prostate-derived Ets transcription factor; PSMA, prostate-specific membrane antigen. (C) Real-time RT-PCR analysis of PR- and GR-target gene expression in LNCaP cells expressing the wild-type PKG-Iβ or mutant PKG-Iβ. The gene expression in cell lines treated with or without 10 nM of progesterone or dexamethasone was determined by real-time RT-PCR, and the fold change  =  gene expression in the presence of hormone/gene expression in the absence of hormone.

Western blot analysis demonstrated that prostate cancer LNCaP cells do not express PKG-Iβ (Fig. 6A, lane 1). We expressed wild-type (lane 2) or D516A mutant (lane 3) PKG1β in LNCaP cells via lentivirus. LNCaP cells expressing the wild-type or mutant (D516A) PKG-Iβ were grown in the absence or presence of 10 nM R1881 and RNAs were prepared for RT-PCR analysis to investigate whether PKG-Iβ regulates endogenous AR-driven gene expression (Fig. 6B). Androgen-mediated inductions of PSA, NKX3.1, prostate-derived Ets transcription factor (PDEF), and p21 were enhanced by wild-type PKG-Iβ expression by about 2.0-, 3.3-, 2.7-, and 1.9-fold, respectively, but were not affected by mutant PKG-Iβ expression. In contrast, PKG-Iβ expression had no effect on c-FLIP, prostate-specific membrane antigen (PSMA), or maspin. Collectively, these results indicate that PKG-Iβ selectively regulates AR-dependent gene expression *in vivo*. Similar analysis indicated that PKG-Iβ also regulates PR and GR-dependent gene expression *in vivo* (Fig. 6C).

### PKG-Iβ expression enhances p44 nuclear localization

**Figure 7:**
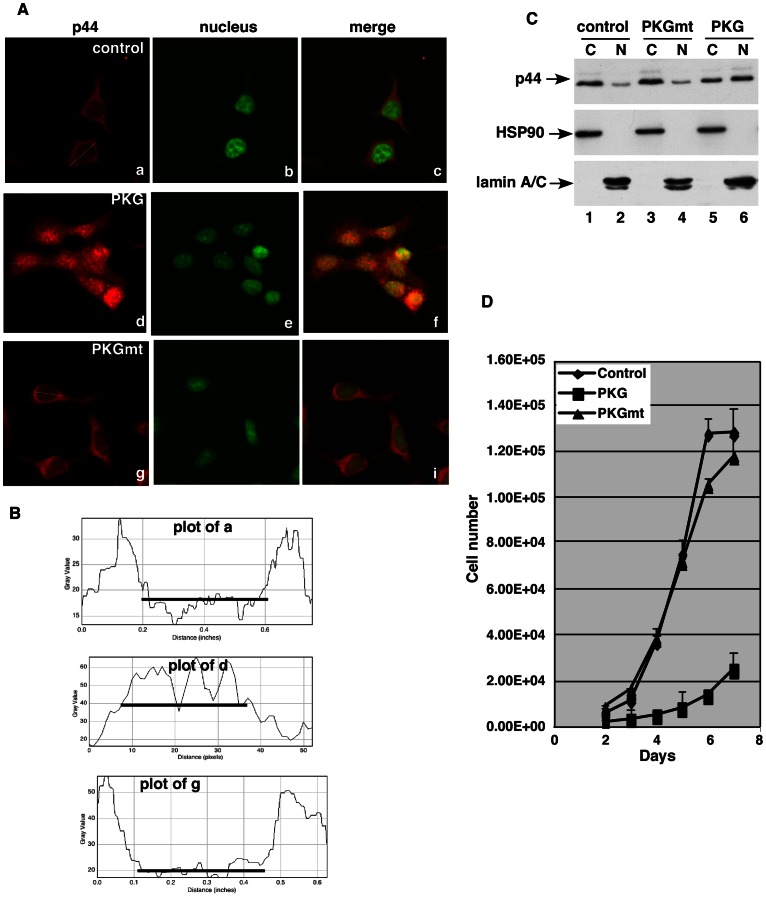
PKGIβ promotes p44 nuclear localization and suppresses LNCaP growth. (A) p44 was detected with immunostaining in control LNCaP cells (top) or LNCaP cells expressing wild-type PKG-Iβ (middle) and mutant PKG-Iβ bottom). P44 was identified with rabbit polyclonal anti-p44 antibody and the Cy5-labeled anti-rabbit immunoglobulin G antibody. SYTOX green was used to stain the nucleus. (B) The fluorescence intensity changes across the white lines (A, left panels) were plotted as the line intensities in the histogram (ImageJ, NIH). The black lines indicate the location of nuclei. (C) Western blot analysis of the cytoplasm (C) or nuclear (N) fraction of the control LNCaP cells (control) or LNCaP cells expressing wild-type (PKG) or mutant (PKGmt) PKG1β with antibodies as indicated. (D) The growth curve of the control LNCaP cells (Control) or LNCaP cells expressing wild-type (PKG) or mutant (PKGmt) PKG1β.

We have previously shown that p44 localization in the nucleus is required for prostate epithelial cell differentiation while its cytoplasm translocation is essential for prostate epithelial cell proliferation and prostate tumorigenesis [44], [45]. Because PKG-1β interacted with and phosphorylated p44, we wonder whether PKG-Iβ would affect p44 subcellular localization. P44 predominantly localized in the cytoplasm in about 70% of control LNCaP sexpressing the mutant PKG-Iβ (Fig. 7A, *andbottom*, 7B *bottom panel*). In contrast, p44 localized in the nucleus in about 70% of LNCaP cells expressing the wild-type PKG-Iβ (Fig. 7A, *middletop panel*, 7B). Western blot analysis of cell cytoplasm and nuclear fractions confirmed this observation (Fig. 7C).

Cell proliferation analysis showed that wild-type PKG-Iβ expression inhibited LNCaP cell growth (Fig. 7D). In contrast, the growth rate of mutant PKG-Iβ cells was not different from that of control LNCaP cells. Flow cytometry analysis of propidium iodide–stained cells revealed that the proportions of PKG-Iβ-expressing LNCaP cells in the G1 phase (54±3%) were significantly higher than that of the control LNCaP (42±5%) and LNCaP cells expressing the mutant PKG1β (40±2%) (Table 1). Conversely, the proportion of PKG-Iβ LNCaP cells in the S phase was 12% lower than the proportion of the mutant PKG-Iβ LNCaP cells in the S phase. Thus, the slow growth of PKG-Iβ expressing LNCaP cells may be attributed to the arrest of cell cycle at the G1 phase, which is consistent with our previous finding that p44 nuclear localization led to G1 cell cycle arrest and inhibited LNCaP cell growth [44], [45]. Our results suggest that the PKG-Iβ enhanced p44 nuclear localization, which in turn suppressed prostate cancer cell proliferation via the G1 cell cycle arrest.

**Table 1:** Cell cycle distribution of control LNCAP or LNCaP.

	Expressing the wild type or mutant PKG1β		
	G1 (%)	G2 (%)	S (%)
Control	42±3	14±0.8	44±4
WT	54±3	13±1	33±2
MT	40±2	15±0.7	45±3

**Figure 8:**
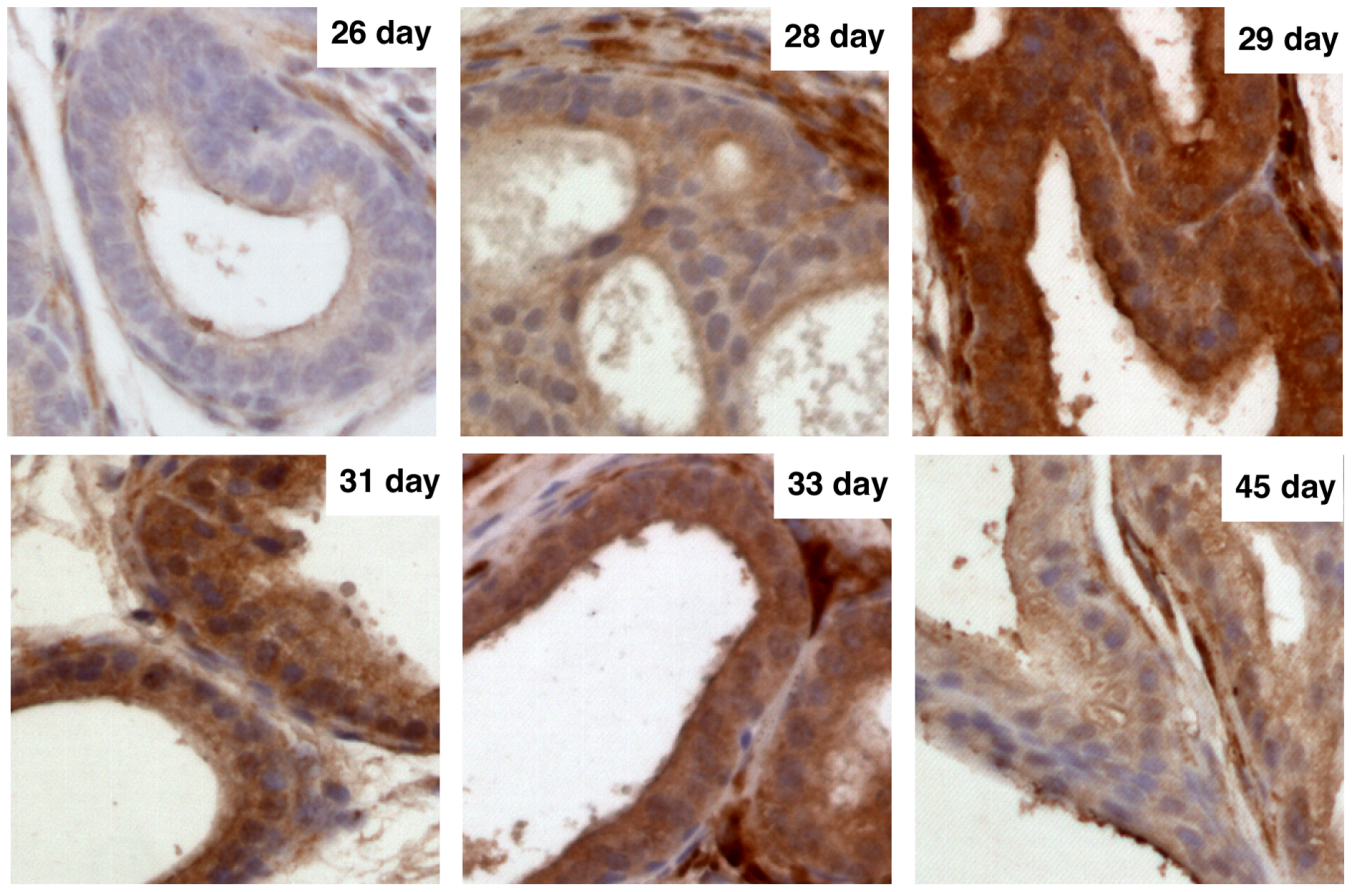
PKG expression is correlated with the p44 nuclear translocation during the mouse prostate development. Prostate glands were dissected from mice at the age of 26, 28, 29, 31, 33, and 45 days and processed for immunostaining with anti-PKG antibody. The p44-expressing cells are stained in brown.

We investigated PKG-Iβ expression during the development of the mouse prostate gland. PKG-Iβ expression was low in epithelial cells during the first 7–26 days after birth (Fig. 8). PKG-Iβ expression was more evident in prostate epithelial cells of mouse at the age of 28 days and dramatically increased in the prostate of mouse aged at 29 days. The PKG expression was then decreased in further aged mouse prostate glands and remained at low but detectable levels in mouse aged at more than 45 days. We previously observed that p44 localized in the cytoplasm of epithelial cells in the prostate of mouse aged at younger than 28 days and began to observe p44 nuclear translocation at the age of 28 days and this process was complete at the age of 45 days [20]. These observations imply that high PKG-Iβ expression might contribute to p44 nuclear translocation during the mouse prostate development.

## Discussion

We found that PKG-Iβ interacts with p44 and regulates AR-driven gene expression. PKG-Iβ expression led to p44 nuclear localization to selectively modulate expression of a set of AR-target genes, thereby leading to G1 cell cycle arrest and suppressing cell growth.

### PKG's roles in regulating AR-driven gene expression

Although PKG has been shown to mediate multiple biological functions in cells [39], [46], little is known about its roles in regulating transcription. Some studies have demonstrated that PKG regulates transcription by cooperating with transcription factors such as AP-1 [47], TFII-I [41], [42], and nuclear factor-kappa B (NF-κB) [48], and that PKG's effect is mediated by sequence elements such as SRE, FAP, CRE [43], and CCAAT/enhancer-binding protein (C/EBP) sites [48]. Although it has been established that PKA signaling activates the AR target gene PSA in the absence of androgen [49]–[52] by activating CREB, which binds to the CRE site in the PSA promoter [49], we are the first to report that PKG regulates the AR signaling pathway.

We found that PKG-Iβ worked in synergy with p44 on AR-mediated transcription, which was consistent with the interaction profile of PKG-Iβ and p44. Also, both PKG-Iβ and p44 [13] enhanced AR-, GR-, and PR-driven gene expression but had no effect on ERα- or TR-mediated transcription, suggesting that PKG-Iβ and p44 may share common machinery in regulating the transactivity of nuclear receptors and they probably always combine in these regulations. Our findings revealed a novel mechanism in activity regulation of nuclear receptors and further investigation into the effect of PKG-Iβ and p44 on AR, GR, and PR would throw light on the mechanical studies of transactivity regulation of nuclear receptors.

PKG has been shown to modulate NF-κB transactivity from distinct recognition sites. PKG dose-dependently increases p65 transactivation on the NF-κB consensus sequence but not on a nonconsensus sequence [48] and enhances the transcriptional activity of p49 or p50 on a unique C/EBP-associated NF-κB site but not on the consensus site. Our study showed that PKG has promoter selectivity and regulates the expression of only a certain group of AR target genes, including PSA, NKX3.1, p21, and PDEF. Thus, PKG seems to recruits to specific promoters it “recognizes” and employs the transcription machinery on them. However, whether these specific sites share common features and how PKG reads those sites remains unclear and further studies are needed.

### PKG physically interacts with and phosphorylates p44

Surprisingly, The PKG-Iβ KD showed stronger interaction with p44 than the full-length PKG-Iβ, indicating an inhibitory mechanism existing inside of PKG-Iβ. Indeed, it has been shown that the N-terminal regulatory domain of PKG-Iβ inhibits C-terminal KD function [35]–[37]. This autoinhibition might prevent the PKG-Iβ KD from interacting with and phosphorylating p44.

PKG-Iβ mediates its intracellular functions by phosphorylating its substrates, TFII-I [41], [42], NF-κB [48], vasodilator-stimulated phosphoprotein [53], and MAPK kinase kinase 1 [54]. In the current study, we demonstrated that PKG-Iβ interacts directly with p44 and found that p44 and AR are substrates of PKG-Iβ. Additionally, PKG-Iβ's kinase activity was required to functionally regulate AR-driven gene expression. Thus, PKG-Iβ may regulate AR transactivation and p44 subcellular localization by directly phosphorylating AR and p44.

### Possible roles of PKG in prostate cancer

Studies have shown that, compared to normal tissue, PKG-Iβ expression is reduced in liver, thyroid, lung, colon, testicular, and pancreatic tumors [55]. PKG activation induces apoptosis in prostate [56]–[60] and colon [61], [62] cancers, induces G1 arrest in vascular smooth muscle cells [63], and inhibits colon cancer cell migration [61], [62]. Sulindac and its derivatives, which are effective in preventing colorectal [58], [64], prostate [60], [65], and breast cancer [66], have been shown to induce cell apoptosis via PKG activation [54], [67]. We observed the absence of PKG-Iβ in LNCaP, PC3, and DU145 cells and that PKG-Iβ expression strongly inhibited LNCaP cell growth. These results are consistent with the well-documented antiproliferation and antitumor effects of PKG.

The translocation of p44 from the nucleus to the cytoplasm is essential to initiate proliferation of prostate epithelium cells [45]; conversely, the nuclear p44 has been shown to promote cell differentiation and inhibit prostate cancer cell proliferation via G1 cell cycle arrest [44], [45]. Our findings indicate that PKG-Iβ expression enhances p44 nuclear localization and G1 cell cycle arrest in LNCaP cells and PKG expression during the mouse prostate development was coincident with the p44 nuclear translocation. These observations strongly suggest that PKG-Iβ regulates p44 subcellular localization to influence the proliferation and differentiation of prostate epithelial cells regulated by the AR signaling pathway, which provide a novel mechanism for the growth inhibition of prostate cancer cells by the androgen signaling. For unknown reasons, PKG-Iβ expression is lost during prostate tumorigenesis, which may lead to p44 cytoplasm localization and prostate epithelial cell proliferation. Clearly, further insight into the molecular mechanisms underlying silencing of PKG-Iβ and cytoplasm localization of p44 in human prostate cancer will help to facilitate the development of therapeutic strategies, involving reexpression of PKG-Iβ and nuclear translocation of p44, for the treatment of prostate cancer.

## Materials and Methods

The MD Anderson Institutional Animal Care and Use Committee approved all the experimental procedures used for mice. Mice were sacrificed by CO_2_ and the prostate glands were dissected for immunostatining.

### Yeast two-hybrid screening

We performed yeast two-hybrid screening as described previously [68]. Briefly (Fig. 1A), we generated an expression plasmid encoding the CytoTrap bait by inserting the cDNA sequences encoding full-length human p44 (amino acid residues 2–342) into pSos, a yeast shuttle vector. *Saccharomyces cerevisiae* (cdc25H), a temperature-sensitive mutant yeast strain that contains a point mutation in the yeast homologue of the hSos gene, cannot grow at 37°C but can grow at the permissive temperature of 25°C. This yeast strain was transformed sequentially with pSos-p44 and a human prostate cDNA expression library fused to the v-Src myristylation sequence (Stratagene, La Jolla, CA), which anchors the fusion protein to the plasma membrane. If the bait and target proteins physically interact, the hSos protein is recruited to the membrane, thereby activating the Ras signaling pathway and allowing the cdc25H yeast strain to grow at 37°C. The clones growing on the plates containing galactose at 37°C were selected as positive clones. We recovered plasmids from each of these positive colonies and identified them by DNA sequencing. The recovered sequence of positive clones was compared with those in the GenBank databases (BLAST, National Center for Biotechnology Information, Bethesda, MD).

### Transient transfection assay

All expression plasmids were generated by inserting the corresponding cDNA sequences into pcDNA3.1. The luciferase reporter plasmid contains four tandem copies of the androgen response element (ARE), the prostate-specific antigen (PSA) gene upstream of the minimal adenovirus E4 promoter [45], [68]–[71]. The luciferase reporters contained the three copies of estrogen response elements (ERE), or two copies of thyroid hormone response elements (TRE) ahead of the E4 basal promoter as described previously [68], [70]. Luciferase reporters containing the MMTV promoter, PSA enhancer (−4354/–3858), probasin (−2441/+28), c-FLIP (−48/+156), and IGFBP5 (−98/+148) were generated as previously described [45], [69]–[71].

PC3 cells were purchased from ATCC (Manassas, VA) and maintained in RPMI1640 medium plus 10% fetal bovine serum. PC3 cells were plated into 24-well plates (1.6×10^4^ cells/well) and transfected 24 h later with 25 fmol of luciferase reporter plasmid, 0.8 fmol of pR-LUC internal control plasmid, and the indicated amounts of expression plasmids. The total amount of DNA was adjusted to 75 fmol with pcDNA3.1. The transfection was conducted with Lipofectamine (Invitrogen, Carlsbad, CA) in serum-free and phenol red-free RPMI 1640 medium. After 6 h, the medium was exchanged for regular RPMI 1640 medium plus 10% fetal bovine serum or phenol red-free RPMI 1640 medium plus 10% charcoal-stripped fetal bovine serum and 10 nM R1881, 10 nM dexamethasone, 10 nM progesterone, 1 µM β-estradiol, or 10 nM T_3_. Cells were cultured for another 48 h and harvested for the dual luciferase assay (Promega, Madison, WI). Three independent experiments were performed for each transient transfection assay.

### Protein-protein interaction assay

For the GST-fusion protein pull-down assay, GST and GST-fusion proteins (1 μg) expressed in bacteria were immobilized in 20 µl of glutathione-agarose beads. The beads were incubated with 5 µl of rabbit reticulocyte lysate that contained ^35^S-labeled proteins in a final volume of 200 µl containing 20 mM HEPES (pH 7.9), 0.2 mM EDTA, 20% glycerol, 2 mM dithiothreitol, 150 or 300 mM KCl, and 0.1% nonyl phenoxylpolyethoxylethanol (NP)-40. The beads were washed with 1 ml incubation buffer five times, boiled in 20 µl of SDS gel sample buffer, and analyzed by SDS-PAGE followed by autoradiography. ^35^S-labeled PKG-Iβ or its domains, including the NTD (amino acid residues 1–355), ATP-binding domain (amino acid residues 355–491), CD (amino acid residues 491–686), and KD (composed of the ATP-binding domain and CD [amino acid residues 355–686]), were incubated with GST or GST-p44 fusion proteins.

For the *in vivo* PKG-Iβ-p44 interaction assay, 3×10^6^ LNCaP cells (ATCC) were transfected with 250 fmol of pcDNA-f:PKG-Iβ or empty vector (pcDNA3.1). After 48 h, transfected cells were harvested, and whole-cell extracts were prepared using passive lysis buffer (Promega). Whole-cell lysates were immunoprecipitated with 20 μl of anti-FLAG M2 agarose beads (Sigma-Aldrich, St. Louis, MO) or anti-p44 antibody immobilized on Protein A Sepharose. The beads were washed 3 times with a buffer containing 20 mM HEPES (pH 7.9), 0.2 mM EDTA, 20% glycerol, 2 mM dithiothreitol, 100 mM KCl, and 0.1% NP-40. The bead-bound proteins were separated by 10% SDS-PAGE. Proteins were transferred to a 0.45-μm Protran nitrocellulose membrane (Whatman, Middlesex, UK), and the membrane was blotted with anti-p44 or anti-PKG (Stressgen, Victoria, Canada) antibody [70].

### *In vitro* kinase assay

AR and p44 proteins were expressed and purified as previously described [68], [70]. PC3 cells (3×10^6^) were transfected with 250 fmol of wild-type pcDNA-f:PKG-Iβ or mutant pcDNA-f:PKG-Iβ (D516A). After 36 h, whole-cell lysates were prepared from transfected cells and incubated with 15 µl of M2 agarose beads for 3 h at 4 C. Beads were washed 5 times with BC300/0.1% NP-40 and then eluted with 30 µl of FLAG peptide (0.2 mg/ml). Wild-type and mutant (D516A) f:PKG-Iβ proteins were checked with SDS-PAGE gel and the gel was stained with Coomassie blue R250. Wild-type or mutant PKG-Iβ proteins (100 ng) were incubated with 80 ng of purified AR and/or 40 ng of purified p44 and 10 µCi of γ-^32^P-ATP in 25 µl of kinase buffer (25 µM 8-Br-cGMP, 10 mM MgCl_2_, 0.5 mM dithiothreitol, 10 mM β-glycerolphosphate, and 25 mM Na-HEPES, pH 7.0) for 1 h at 30 C. The reaction mixtures were analyzed by SDS-PAGE and the phosphorylated proteins were visualized by autoradiography.

### Expression of PKG-Iβ in LNCaP cells

cDNAs of wild-type or D516A The mmutant PKG-Iβ was subcloned into the lentiviral expression vector (dsRed-OG2). The recombinant lentivirus was produced with 293T as described [72]. To express PKG-Iβ, LNCaP cells (1×10^5^) were plated in 6-well plates and transduced with the virus containing empty vector, wild-type PKG-Iβ, or mutant PKG-Iβ. After 48 h, the cells were replated and the PKG-Iβ expression was confirmed by Western blot with anti-PKG polyclonal antibody.

### Real-time RT-PCR analysis

Real-time RT-PCR was performed as described previously [45]. Briefly, cells were grown in phenol-free RPMI 1640 medium plus 10% charcoal-stripped fetal bovine serum for 3 days and treated with or without 10 nM R1881 for 24 h. Total cellular RNAs were isolated from cells using an RNeasy Protect mini kit (Qiagen Inc., Valencia, CA), and cDNA was synthesized by reverse transcription using a Reaction Ready first strand cDNA synthesis kit (SuperArray Bioscience, Frederick, MD). The resultant cDNA product was subjected to PCR amplification with an RT^2^ SYBR Green real-time PCR master mix and gene-specific primer sets using a SmartCycler II (Cepheid, Sunnyvale, CA) under the following conditions: 40 cycles of 30 sec at 94°C; 20 sec at 55°C; 30 sec at 72°C. RT-PCR primer sets were purchased from SuperArray Bioscience. The raw data of the PCR reaction were processed and quantified with SmartCycler Software version 2.0c (Cepheid). Three independent analyses were performed for each sample, and changes in expression of AR target genes induced by R1881 were calculated with the 2^ΔΔCt^ method [73].

### Immunofluorecent staining

LNCaP cells expressing wild-type or mutant PKG-Iβ were fixed in 3% paraformaldehyde. The antigen retrieval and immunohistochemical staining were performed as described (29). A rabbit polyclonal anti-p44 antibody was applied to the LNCaP cells at a 1∶100 dilution and the FITC-labeled anti-rabbit immunoglobulin G antibody. Hoechst was used to locate the cell nucleus. The fluorescent signals were observed under a fluorescent microscope.

### Cell proliferation assay

LNCaP cells expressing wild-type or mutant PKG-Iβ were plated in a 24-well plate at 5×10^3^ cells/well and counted every day for 6 days.

### Mouse prostate separation and immunostaining

The male C57/B6 mice (n = 16) were sacrificed at the age of 26, 28, 29, 31, 33, and 45 days, the prostate glands were removed en bloc (including the seminal vesicles, urethra, and bladder) and fixed with 4% paraformaldehyde in PBS at 4°C overnight and embedded in paraffin. Sections (4 µm) were cut and mounted on Super-frost Plus adhesion slides (Fisher). Anti-p44 (1∶500) and anti-PKG (1∶1,000) were applied to the prostate tissue sections and incubated overnight. A streptavidin-biotin peroxidase detection system was used according to the manufacturer's instructions (DAKO A/S, Grostrup, Denmark), with 3, 3′-diaminobenzidine as substrate. The MD Anderson Institutional Animal Care and Use Committee approved all the experimental procedures used for mice. The MD Anderson Institutional Animal Care and Use Committee approved all the experimental procedures used for mice.
